# An Inquiry into the Nature and Cause of That Swelling, in One or Both of the Lower Extremities, Which Sometimes Happens to Lying-In Women. Together with an Examination into the Propriety of Drawing the Breasts, of Those Who Do, and Also of Those Who Do Not Give Suck

**Published:** 1784

**Authors:** 


IV. *An Inquiry into the nature and cauſe of that ſwelling, in one or both of the lower extremities, which ſometimes happens to lying-in women. Together with an examination into the propriety of drawing the breaſts, of thoſe who do, and alſo of thoſe who do not give ſuck. By* Charles White, *Eſq.* F. R. S. *Member of the Corporation of Surgeons in London; Surgeon to the Infirmary and Lunatic Hoſpital, and Vice President of the Literary and Philoſophical Society of Mancheſter, &c.* 8vo. Dilly, *London,* 1784. 87 pages, with 3 copper-plates.

BEFORE the practice of midwifery fell into the hands of men of ſcience, effects were too often miſtaken for cauſes, and the diſorders of lying-in women were in general aſcribed to ſome defect, redundance, or ob-

ſtruction

ſtruction of the lochia, or of the milk. This has been the caſe with that which is the ſubject of the preſent inquiry. It has been attributed to ſuppreſſions of the lochia, to depoſits or redundancies of milk, or to cold, and has been confounded with other diſorders. But the ingenious author of the work now before us undertakes to prove that it is a diſorder *ſui generis*, and proceeds from a cauſe not hitherto ſuſpected.

The French are the principal authors who have written upon this ſubject; but their deſcription of the diſorder, we are told, is incorrect, and their mode of treatment inadequate. Mauriceau, who is the firſt author who has deſcribed it, aſcribes it to a reflux of lochia on the part; but Puzos, Levret, and in general all the later French writers, together with Baron Van Swieten, conſider it as a depoſit of milk.

Mr. White has found but little account of this complaint in any Engliſh author, and he obſerves that it is but ſlightly mentioned by the lecturers on midwifery either in London or Edinburgh. Dr. Hunter, it ſeems, uſed to ſpeak of it in his lectures, but without determining its real nature; and Dr. Denman has been accuſtomed to conſider it as an affection of the whole glandular and lymphatic fyftem of the extremity.

The fymptoms of this diforder are defcribed with great accuracy by our author. In one cafe he has feen it occur fo early as twenty-four hours after delivery; and in another fo late as five weeks; but neither of thefe periods of attack, we are told, is ufual, as in general it comes on about the fecond or third week after delivery. The firft fymptom is commonly a pain in the groin. This part foon becomes affected with fwelling and tenfion, which extend to the labium pudendi of the fame fide, and down the infide of the thigh, to the ham, the leg, the foot, and the whole limb; and the progrefs of the fwelling is fo quick, that in a day or two the limb becomes twice the fize of the other, and is moved with great difficulty, is hot and exquifitely tender; but not attended with external inflammation. In a few days the heat and pain abate, but the fwelling and fome degree of tendernefs continue feveral weeks. The fkin is tenfe, pale, and fhining, but does not yield to preffure as in anafarca, neither does any water iffue from it on its being punctured with a lancet.

The fwelling comes on without rigor or fhivering, but is ufually attended with a fmall fever, which which in ſome patients ſubſides in two or three weeks, but in others continues ſix or eight weeks, and is attended with hectic ſymptoms. Sometimes, tho' rarely, we are told, the ſwelling attacks both the lower limbs. It is not uncommon, however, it ſeems, after the diſorder has ſubſiſted a week or two, for the ſound leg to become œdematous towards evening; but then the groin and thigh are not affected on that ſide; and the leg is much ſofter to the touch than the other, and pits when preſſed by the finger.

This diſorder, the author obſerves, attacks indifferently women of all ranks and of different ages and habits; thoſe who give ſuck and thoſe who do not; the ſtrong and the weak; after their firſt or any other labour; and whether the labour be natural or preternatural; but he has not known it happen after a miſcarriage, nor to a woman more than once tho' ſhe has afterwards had more children. It happens at all ſeaſons of the year indiſcriminately; and in the country, as well as in large towns. He has never ſeen it attack any other parts of the body, except thoſe already mentioned; nor has he ever ſeen it ſuppurate, or prove fatal, or occa-

ſion

ſion any other inconvenience, after a few months, than a little ſwelling of the leg after fatigue.

This general hiſtory of the diſeaſe is illuſtrated by a variety of caſes. The author then proceeds to deliver his theory concerning its nature and cauſe ; but before he attempts to aſcertain what it is, he has thought it right to ſay what it is not, by pointing out its difference from other diſorders to which it bears ſome reſemblance. This leads him to mention the circumſtances in which it differs from ſciatica, rheumatiſm, anaſarca, and eryſipelas. It cannot be owing, he obſerves, to any defect of the lochia, as it happens to thoſe who have the moſt regular diſcharge ; nor can it be in any meaſure owing to the milk, as it occurs under every circumſtance attending that ſecretion, and under every different treatment of the breaſts. It cannot be an affection of the whole lymphatic ſyſtem, as it is confined to the lower extremities ; and further, Mr. White contends that it cannot be properly called a diſeaſe of the arteries, veins, nerves, muſcles, or bones, as it is not accompanied with any ſymptom attendant upon diſorders of thoſe parts. From theſe premiſes, and from the hiſtory he has given of the diſeaſe, he thinks we may conclude that its proxiproximate cauſe is an obſtruction, detention, and accumulation of the lymph in the limb. This obſtruction, he thinks, may be aſcribed to the preſſure which the lymphatics, in their paſſage over the brim of the pelvis, experience from the head of the child during labour. The current of lymph, thus ſtopped, is prevented from regurgitating by the number of valves with which the abſorbents are furniſhed, and of courſe a lymphatic ſo compreſſed will be diſtended till it burſts. The fluid thus extravaſated will be abſorbed by other lymphatics, and in the mean while the ruptured veſſel will be gradually reunited. If the above hypotheſis be true, he thinks the prediſponent cauſe may, in all probability, be a weakneſs of the coats of the lymphatics, in ſuch ſubjects only as have theſe veſſels formed into one principal trunk under Poupart's ligament. — Such are the outlines of our author's theory, and to elucidate it he has introduced a deſcription of the lymphatics of the lower limbs from the lateſt and beſt writers on the ſubject; and, with permiſſion of Mrs. Hewſon, has decorated his work with the three firſt plates of Mr. Hewſon's work on the lymphatic ſyſtem.

For

For the cure of this diſeaſe the author, in the firſt, or what he thinks may be called the inflammatory ſtage, recommends the antiphlogiſtic method. But as the inflammation here is not the original complaint, but only a ſymptom, he prudently cautions us againſt waſting our patient's ſtrength by large evacuations. Glyſters and mild purges, he obſerves, will be ſufficient. To alleviate the pain he adviſes the uſe of opiates, anodyne fomentations, the warm and vapour baths, and bliſters applied to the upper part of the thigh; theſe laſt, he tells us, have generally been found of much advantage, by taking off the irritation from the part originally affected. The fever is to be alleviated by antimonials, the neutral draughts, cool acidulated liquor, and cool air; and if the lochia are acrid and putrid, antiſeptic injections are to be thrown up the vagina with a large ivory ſyringe or by an elaſtic vegetable bottle.

When the violence of the pain abates, and the ſwelling and tenſion begin to leſſen, but a quick pulſe and ſome degree of fever remain, the diſeaſe is ſaid to have arrived at its ſecond ſtage. At this period he recommends wine and a fuller diet, and has often experienced good effects from calomel in ſmall doſes, and likewiſe from from myrrh. The limb may now be chafed with warm oil, and the patient is advifed to ufe the tepid bath. When the pain and fever are entirely gone, and no complaint remains except a fwelling of the limb, and perhaps a general relaxation, the bark, with or without fteel, fea-bathing, or the cold bath, dipping the limb in cold water, embrocating it with camphorated fpirit of wine or with diftilled vinegar, a ftrait ftocking or fome other fuitable bandage, exercife on horfeback, and gentle rubbing of the limb, are the methods recommended to complete the cure.

In an appendix the author defends an opinion he had given in a former work relative to the drawing the breafts of women, againft Mr. Cruttwell of Bath, who denies the neceffity or even propriety of ever doing it. But when from the weaknefs of the child, or the bad conformation of the nipple, or other accidental circumftances, it is impoffible or improper for the child to fuck, there furely can be no objection to our continuing to folicit the milk by this method till the impediments in queftion are removed; and thefe are the only cafes in which its ufe is infifted on by Mr. White.

